# Liquid biopsy for monitoring medulloblastoma

**DOI:** 10.20517/evcna.2022.36

**Published:** 2022-09-28

**Authors:** Robert H. Eibl, Markus Schneemann

**Affiliations:** ^1^c/o M. Schneemann, Department of Internal Medicine, Hospitals of Schaffhausen, 8208 Schaffhausen, Switzerland.; ^2^Department of Internal Medicine, Hospitals of Schaffhausen, 8208 Schaffhausen, Switzerland.

**Keywords:** Medulloblastoma, monitoring, CSF, cell-free DNA, cfDNA, circulating tumor DNA, ctDNA, biomarker, liquid biopsy, brain tumor

## Abstract

Despite recent progress in molecular diagnostics defining four distinct medulloblastoma groups, the clinical management of these malignant childhood tumors of the cerebellum remains challenging. After surgical removal of the tumor, both cytotoxic chemotherapy and irradiation can offer additional curative benefits, but they also include a significant risk of long-term damage. Early molecular profiling aims to predict the outcome of such aggressive therapies. This prevents unnecessary damage to patients who may not need it and helps to identify those patients with remaining tumor cells who may benefit from more aggressive treatment with the intent to cure. Monitoring tumor evolution in real time allows personalized precision medicine with an immediate clinical response resulting in a better outcome. Liquid biopsy includes various methodologies already applied in numerous studies and clinical trials for common cancers including brain tumors, but information on medulloblastomas is limited. This review summarizes the recent developments of how liquid biopsy can support or even replace the standard monitoring of medulloblastomas by medical imaging or cytology and discusses what will be needed to make liquid biopsy a new gold standard in diagnosis, therapy, and follow-up of medulloblastomas for the benefit of the patients.

## INTRODUCTION

Medulloblastoma is the most common malignant brain tumor in children, but it can also develop in younger adults. As the father of modern neurosurgery, Harvey Cushing significantly reduced mortality by inventing better neurosurgical procedures a century ago. With Parcival Bailey, he also coined the term medulloblastoma for this histological entity of posterior fossa tumors [Table 1]^[3]^. Cushing tried local irradiation after surgery, but a major breakthrough with this technique was developed only later; when in 1953 not only the brain but the whole developing CNS including the spinal cord was irradiated to prevent metastatic growth. In the 1980s, cytotoxic chemotherapy was added. Unfortunately, both aggressive chemotherapy and irradiation can lead to severe damage to the CNS as well as, rarely, to secondary tumors^[25]^. To avoid over- and undertreatment, it is tempting to identify those patients in advance who may benefit with intent to cure and those who may not need it. Therefore, it was important to realize that medulloblastoma *per se* does not exist, or it does not define such a homogeneous group of tumors as the name suggests. The term medulloblastoma still summarizes morphologically similar but biologically heterogeneous tumors of the cerebellum. The cell of origin remains unclear, but an embryonal origin is supported by single-cell sequencing studies and the peak incidence in early childhood^[9,26-30]^.

**Table 1:** Historical timeframe and developments leading to liquid biopsy of medulloblastomas

**Year**	**Author**	**Probe**	**Method**	**Tumor**	**Milestone**
1868	Ashworth^[1]^	CTC	Microscopy, case report	Skin metastasis of unknown primary tumor, “liquid autopsy”	First report on tumor cells in blood; post mortem; microscopically identical cells in metastatic lesions
1889	Paget^[2]^	CTC	Autopsy	Breast cancer, postulated	Seed and soil “theory of cancer metastasis”
1925	Bailey and Cushing^[3]^	Neurosurgically removed posterior fossa tumors	Histology	Medulloblastoma	Introduced the name medulloblastoma
1948	Mandel and Métais^[4]^	cfDNA	Blood analysis	Not related to cancer, healthy blood donors	First report of (cell-free) nucleic acids in blood
1953	Paterson and Farr^[5]^		Irradiation: 5000 cGy posterior fossa 3500 cGy neuraxis	65% 3-year survival	Irradiation treatment of the whole CNS
1975	Fidler^[6]^	CTC	Experimental metastasis assay	B16 melanoma cell lines	Only a small fraction of intravenously injected tumor cells give rise to metastasis in mouse models
1977	Leon *et al.*^[7]^	cfDNA	Radioimmunoassay for free DNA in serum	Various cancers	First report on increased DNA levels in some cancer patients; correlation with therapy response
1991	Eibl and Wiestler^[8,9]^	Experimentally induced tumors and derived cell lines	Retrovirus-mediated gene transfer of SV40 LT into neural transplants	PNET	Rat tumor model, histologically identical to human medulloblastomas (neuroblastic rosettes, bipotential differentiation), triggered medulloblastoma research in Germany
1991	Ohgaki, Eibl *et al.*^[10]^	Primary tumor tissue	SSCP-PCR, direct sequencing	Medulloblastoma	First detection of p53 mutations in primary medulloblastoma tissue by Eibl, supporting Eibl’s earlier tumor model of inactivation of p53, also triggered medulloblastoma research
2001	Reya *et al.*^[11]^	CTC	Applying hematopoietic stem cell knowledge to the heterogeneity of cancer cells, self-renewal	Solid tumors and leukemia, migratory CSC	Cancer stem cell theory (Weissman/Clarke)
2003	Balaña *et al.*^[12]^	ctDNA	Methylation-specific PCR of MGMT, p16, DAPK, RASSF1A	GBM	Detection of methylated MGMT in serum highly predictive for response to BCNU chemotherapy
2004	Allard *et al.*^[13]^	CTC	CellSearch™	Prostate, breast, ovarian, CRC, lung, and other cancers	Detection of CTCs in 7.5 mL of blood samples
2004	Cristofanilli *et al.*^[14]^	CTC	CellSearch™ Amount of CTC	Metastatic breast cancer	Independent predictive marker: reduced PFS and reduced OS
2010	Pantel and Alix-Panabières^[15]^	CTC	Concept of analyzing tumor cells in body fluids	All cancers	Coined the term “liquid biopsy”
2014	Bettegowda *et al.*^[16]^	ctDNA	Digital PCR, sequencing	14 tumor types	ctDNA detectable for most tumors outside brain
2014	Sullivan *et al.*^[17]^	CTC	“Negative depletion” CTC-iChip (removing leukocytes from blood)	GBM (usually not metastatic)	Surprising and frequent detection of CTCs in brain tumors
2016	Louis *et al.*^[18]^	Tissue biopsy	Molecular profile	Medulloblastoma	New WHO classification, introducing four new medulloblastoma groups based on molecular genetics
2016	Donaldson and Park^[19]^	ctDNA	Clinical studies	NSCLC	First FDA^[20]^ and EMA approval to use ctDNA for EGFR-targeted therapy
2018	Garzia *et al.*^[21]^	CTC	Parabiotic xenograft model	Medulloblastoma	Discovery of a hematogenous route of metastasis to leptomeninges by CCL2-CCR2 axis
2018	Cohen *et al.*^[22]^	ctDNA, plus proteins from blood	CancerSEEK, detecting mutations in 1933 loci of 16 genes; combined with protein tumor markers	8 cancer types	Blood screening test for several common cancers
2020	Lennon *et al.*^[23]^	ctDNA, protein markers plus PET-CT	Prospective 16 gene locations, 8 tumor proteins, PET-CT	Multi-cancer screening of 10,000 women with no known cancer	Multi-cancer blood testing combined with PET-CT
2021	Louis *et al.*^[24]^	Tissue biopsy	Molecular profile, incl. methylation profile	Medulloblastoma	Newest WHO classification, four molecular groups further defined by methylome; additional subgroups (4 SHH; 8 non-WNT/non-SHH)

CRC: Colorectal cancer; CSC: cancer stem cell; CTC: circulating tumor cell; ctDNA: circulating tumor DNA; EMA: European Medicines Agency; FDA: US Food and Drug Administration; PCR: polymerase chain reaction; PET-CT: positron emission tomography-computed tomography; SSCP: single-strand conformation polymorphism.

Meanwhile, it became clear that medulloblastomas are genetically distinct from primitive neuroectodermal tumors (PNET), although they share histologically indistinguishable characteristics such as tumor cell morphology, neuroblastic rosettes, and bipotential differentiation with the expression of glial and neuronal markers. A rat tumor model for PNETs developed by Eibl and Wiestler^[8,9]^, using gene transfer of SV40LT to inactivate tumor suppressor genes including TP53, also triggered the detection of the first TP53 mutations in medulloblastoma biopsies by Eibl three decades ago^[10] ^[Table 1]. Others were unable at that time to detect such TP53 mutations in tissue biopsies or in xenografts of human medulloblastomas, except in only one cell line^[31]^; however, this mutation has been considered a common selection artifact during cell culture. The continued diagnostic and prognostic application of TP53 mutations in medulloblastomas supported further genetic profiling and helped to develop the current tumor classification^[8-10,32-41]^: only recently, in 2016^[18]^ and with an update in 2021^[24]^, the World Health Organization (WHO) introduced four new diagnostic groups of this childhood brain tumor based solely on molecular genetic features [Table 2].

**Table 2:** Molecular classification of four groups of medulloblastomas according to the WHO

**Medulloblastoma, molecularly defined**	**Pathway**
Group 1	WNT-activated
Group 2	SHH-activated and TP53-wildtype
	SHH-activated and TP53-mutant
Group 3	(non-WNT/non-SHH)
Group 4	(non-WNT/non-SHH)

SHH: Sonic hedgehog; WHO: world health organization; WNT: wingless/integration-1.

The correlation between different biological behavior and personalized risk assessment may prevent harmful radiation and chemotherapy when unnecessary or not useful. The first two groups refer to different oncogenic signaling pathways: (1) wingless/Integration-1 (WNT)-activated; and (2) Sonic Hedgehog (SHH)-activated. WNT-activated medulloblastomas show the highest five-year survival and a low prevalence of metastatic disease. SHH-activated medulloblastomas can be further separated into two different subgroups, TP53-mutant or TP53-wildtype. SHH-activated and TP53-mutant occur primarily in older children and have a very poor prognosis, whereas SHH-activated and TP53-wildtype, which are most common in adolescents and young children, have a good prognosis. The other two groups are non-WNT/non-SHH, Group 3 and Group 4, respectively. Group 3 shows an increased prevalence of metastatic disease with the poorest five-year survival, whereas Group 4 has an increased prevalence of metastatic disease with a moderate five-year survival. TP53 mutations in SHH medulloblastomas are associated with poor survival and treatment failures^[18]^. Several subgroups have been associated with TP53 and other mutated genes: for WNT-activated, CTNNB1 and APC; for SHH-activated, TP53, PTCH1, SUFU, SMO, MYCN, and GLI2 (methylome); and, for non-WNT/non-SHH, MYC, MYCN, PRDM6, and KDM6A (methylome). Since the WHO classification suggests that the diagnosis from molecular profiling of a tissue biopsy is superior to that of classical histopathology, at least for brain tumors, it is tempting to apply ctDNA-based liquid biopsy for monitoring such mutations in brain tumor patients to avoid repeated and troublesome surgical biopsies. Newer studies successfully used panels of genes.

For brain tumors including medulloblastomas, CSF offers another chance to find ctDNA with a higher sensitivity than plasma or serum^[42-46]^. ctDNA from CSF represents the genomic mutations better than plasma, and CSF shows an increased sensitivity for putative actionable mutations and CNA (copy number aberrations; EGFR, PTEN, ESR1, IDH1, ERBB2, and FGFR2)^[47]^. This improves prognostic evaluation, therapy decisions, and monitoring of treatment, e.g. irradiation, chemotherapy, and future immune therapies.

**Figure 1:**
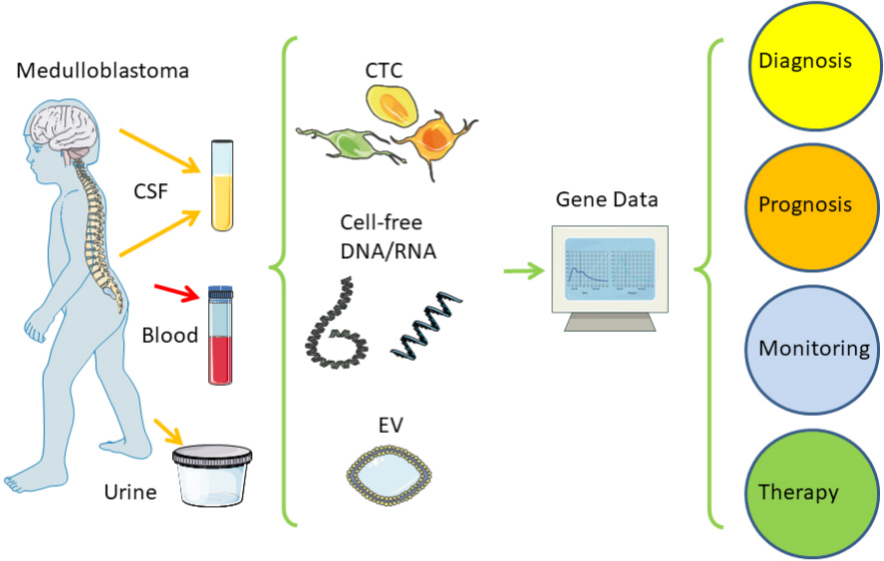
Liquid biopsy of medulloblastomas. Distant to the cerebellum, body fluids such as blood, CSF, or urine can be taken at low risk and then analyzed for relevant genetic information from the childhood brain tumor to support clinical decision making. CSF: Cerebrospinal fluid; CTC: circulating tumor cell; EV: extracellular vesicle. Created/modified with SMART^[48,49]^.

It is reasonable to apply this molecular expertise from classical tissue biopsy to liquid biopsy in order to improve the monitoring of tumor evolution and response to treatment, as well as to avoid elaborate surgical biopsies with a higher risk of neurological or infectious complications. Tumor-derived, cell-free nucleic acids (cfDNA/RNA) and extracellular vesicles (EV) can be found outside of the original tumor in body fluids, such as blood, cerebrospinal fluid (CSF), peritumoral cysts, and urine. Surprisingly, even intact circulating tumor cells (CTC) can be found in blood and CSF [Figure 1]. Analysis of cell-free nucleic acids allows improved and personalized monitoring of patients to adapt rapidly to new therapy decisions without the higher risk of neurosurgical tissue biopsies. Here, we provide an overview of recent developments without an emphasis on technological methods and details, but with the clinical application potential, as well as current limitations and challenges in how future standards need to be developed in order to improve the clinical management of medulloblastomas within the next few years.

## LIQUID BIOPSY

Within the past two decades, different methodologies have evolved that can be summarized as liquid biopsy [Figure 1]^[50,51]^. In principle, tumor-derived material, including whole cells or parts thereof such as nucleic acids and extracellular vesicles (EV), can be detected at locations quite distant from the original tumor or its metastases. This can often be achieved with easy and less risky access than a classical surgical tissue biopsy. Whereas blood tends to be the biofluid of choice for many other cancers, CSF appears to be more suitable for brain tumors. This is partly due to the blood-brain barrier (BBB), preventing cells from entering the bloodstream. CSF also offers less background in terms of leukocytes or cell-free DNA compared to blood, resulting in a better signal-to-noise ratio for many analytical methods. Many medulloblastoma patients develop hydrocephalus which is commonly drained before tumor removal. This can also be an easy source for obtaining CSF, in addition to standard lumbar puncture for follow-ups. The historical timeframe for the development of liquid biopsy and medulloblastoma research is shown in Table 1.

### ctDNA

**Figure 2:**
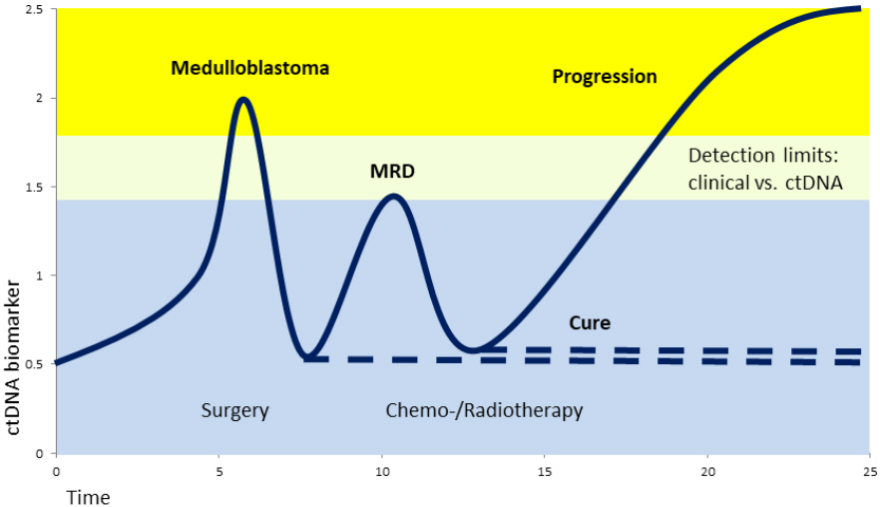
Scheme of ctDNA biomarker level during medulloblastoma development, therapy, and progression. Sequential analysis of CSF supports diagnosis and early detection of minimal residual disease (MRD) as well as clinical decisions for the best benefit of the patient. CSF: Cerebrospinal fluid.

ctDNA is a varying part from much less than 1% to 10% of the total cell-free DNA. Individual changes in ctDNA amounts often correlate with tumor development [Figure 2]^[50]^. An increase of ctDNA can point to metastatic progression, whereas a reduction of ctDNA indicates a treatment response. No reduction of ctDNA after treatment indicates a lack of response. A later increase after an initial decrease indicates resistance development. More diagnostic and prognostic information comes from sequence analysis, which can be targeted or non-targeted to detect mutations or epigenetic signatures of methylation in the tumor.

### CTCs, EVs, miRNA, circRNA and other biomarkers

In 2004, the detection of CTCs with CellSearch was approved for clinical use to detect and count the number of CTCs per blood sample. This detection system uses an epithelial marker to select carcinoma cells, but this marker is not present in brain tumors. Other methods or modifications need to be used and further developed to reach the required sensitivity on brain tumors. Since CTCs are extremely rare, it was surprising to detect CTCs in the blood of glioblastoma patients^[17,52-57]^, an aggressive brain tumor usually found in adults. In 2018, Garzia and colleagues were able to detect CTCs in the blood of medulloblastoma patients^[21]^. Those patient-derived CTCs were able to spread in a xenograft model via the blood to form leptomeningeal metastases, thus questioning the general assumptions of medulloblastomas spreading only, or preferentially, via the CSF. A chemokine highly expressed on medulloblastoma cells, CCL2, was identified with its receptor CCR2 to drive this leptomeningeal homing. Similar potential mechanisms of organ-specific metastasis involving chemokines and their receptors, thus mimicking lymphocyte homing, have been investigated, including at the single-molecule level, with atomic force microscopy^[58-65]^, but not yet with medulloblastoma cells.

Tumor and normal cells can release small, extracellular vesicles (EVs), which protect their included proteins and different sorts of nucleic acids. Recently, EVs from blood, urine, or tissue samples have sparked great interest in liquid biopsy. EVs collected from CSF appear to be superior due to the reduced number of EVs from leukocytes compared to blood^[66]^. EVs from urine can also be used to obtain EV-encapsulated marker candidates with a nanowire scaffold, even from non-urologic cancers^[67]^.

MicroRNAs (miRNA, miR) are 20-24 nucleotides long, non-coding RNA molecules with regulatory and stabilizing effects of translating mRNA. miRNAs seem to play a role in tumor biology, angiogenesis, and immunology. Although their functions are not fully understood, they can serve as markers or potential therapeutic targets in glioblastomas^[68]^. In a diagnostic model, urinary miRNA detection was able to confirm different CNS tumors, including neuroblastoma, with high sensitivity and specificity^[69]^.

Circular RNAs (circRNAs) consist of a closed loop without polyadenylation signal. They appear to be more stable than miRNAs and seem to be similarly involved in gene regulation, although their functions are not well understood. Many circRNAs may just serve as a sponge for miRNAs, thus inactivating the function of a specific miRNA. Due to their stability, they may serve as candidate markers for disease, e.g. circ_463 for medulloblastomas^[70]^.

Proteomic analyses from blood, CSF, or urine may also reveal protein-based biomarkers for screening and monitoring of medulloblastomas in the future. Recently, bioinformatics allowed discriminating medulloblastoma patients from healthy individuals by analyzing a combination of potential protein biomarkers in urine samples^[71]^.

Many studies have been developed by academic collaborations leading to publications that offer significant access to data for reproducibility, metanalysis, and data mining. Large clinical studies usually have major industrial findings and may have more restrictions for sharing data, but they may also eventually allow at least partial access to data. As in many developing fields, there is a demand for data to satisfy the FAIR principles of “findable, accessible, interoperable, and reusable data”^[72]^, which may help to compare different studies.

## CLINICAL STUDIES

Few studies on medulloblastomas have been developed recently in the field of liquid biopsy with varying levels of success, but they basically confirm CSF as currently the most suitable source to analyze ctDNA, followed by blood and urine [Table 3]. Unfortunately, the challenges of repeatedly isolating sufficient amounts of ctDNA even from CSF appear to be high, thus reducing the sensitivity and applicability for many medulloblastoma patients. Furthermore, the low number of mutations in medulloblastomas poses a source for artifacts and needs further evaluation. More clinical studies are needed to establish suitable standards. Currently, ctDNA as a routine marker for tumor monitoring appears to be useful mainly for subsets of medulloblastomas, i.e. progressed high-grade tumors, those with a close connection to the CSF, or for larger children or adult patients with easier access to sufficient amounts of CSF and blood.

**Table 3:** Studies using ctDNA or ctRNA from CSF for screening or monitoring medulloblastomas

**Year**	**Author**	**Tumor**	**Method**	**Findings**
2020	Escudero *et al.*^[73]^	MB	WES, CNVs	ctDNA from CSF sufficient for diagnosis of MB-subgroups, risk stratification and monitoring (proof of concept study)
2020	Li *et al.*^[74]^	Pediatric MB	Whole genome methylation sequencing	High specificity and sensitivity to monitor treatment response of epigenetic signatures in ctDNA from CSF, potential diagnostic and prognostic value
2021	Liu *et al.*^[75]^	MB	WGS	ctDNA from serial CSF samples as prospective marker for MRD, in half of the patients before radiographic progression
2021	Sun *et al.*^[76]^	Pediatric MB	Deep sequencing/NGS, ctDNA in CSF	More alterations detectable in ctDNA from CSF than from primary tumor, superior monitoring technique when ctDNA is detected from CSF
2022	Lee *et al.*^[70]^	MB	RT-PCR sequencing	Circular RNA circ_463 as a candidate biomarker
2022	Pagès *et al.*^[77]^	Pediatric CNS tumors, incl. MB	ULP-WGS, deep sequencing of specific mutations and fusions	ctDNA is detectable better in CSF than blood, not in urine. Molecular profiling is feasible for a small subset of high-grade tumors (incl. MB). Liquid biopsy remains a major challenge for such tumors with low clonal aberrations
2019-2024	NCT03936465^[78]^ ongoing Phase I study, 66 patients	Pediatric cancer, incl. brain tumors	ctDNA	Clinical toxicity study; ctDNA markers in blood and CSF planned as a response to treatment

CNV: Copy number variation; CSF: cerebrospinal fluid; MB: medulloblastoma; MRD: minimal residual disease; ULP-WGS: ultra-low-pass whole-genome sequencing; WES: whole exome sequencing.

As a proof-of-concept study, Escudero and colleagues showed that ctDNA from CSF can provide valuable information about diagnosis and prognosis^[73]^: the genomic alterations represent and characterize the heterogeneity of the tumor and allow the identification of medulloblastoma subgroups and subtyping with risk stratification. In all cases, the CSF was negative from the cytologic analysis, i.e. no intact tumor cells were detectable. Prior to the analysis of ctDNA, somatic mutations from matched tumors were detected with WES and then validated for ddPCR. Before surgery, CSF-ctDNA was detectable in 77% of patients, but not in the plasma, except in 1 of 13 patients. This sensitivity demonstrates the feasibility and superiority of CSF-ctDNA above both cytology from CSF and ctDNA from plasma. CSF-ctDNA monitoring can identify the minimal residual disease (MRD) and genomic tumor evolution.

Since oncogenic mutations in medulloblastomas appear to be much less frequent than those in most other tumors, Li and colleagues used a different approach for tumor monitoring of serial CSF samples^[74]^. Epigenetic changes were reliably detected by whole-genome methylation sequencing (WGMS). DNA methylation as well as hydroxymethylation profiles from CSF matched the signatures from the original tumors in the same patients, thus allowing ctDNA to be used in monitoring treatment response and tumor recurrence. High sensitivity to detect MRD was shown in serial samples after treatment, even when the cytology was negative. The high specificity and sensitivity of these epigenetic signatures from CSF samples may be used for diagnostics and prognosis.

In a prospective trial, Liu and colleagues confirmed the clinical utility of CSF-ctDNA with 476 serial samples from 123 children with medulloblastoma^[75]^. Low-coverage whole genome sequencing allowed the detection of 54% of localized and 85% of metastatic disease cases at baseline. Response to therapy is shown by a reduction of ctDNA, whereas persistent detection after therapy points to a higher risk of progression. ctDNA as a surrogate marker for MRD can detect tumor progression earlier than MRI or CSF cytology. Primary tumors located adjacent to the CSF reservoir allowed Sun and colleagues to isolate and investigate ctDNA from 15 out of 58 patients with medulloblastoma^[76]^. Alterations between primary tumor and CSF-ctDNA are shared, but more alterations were detected in the CSF-ctDNA, which may reflect the evolution of the tumor as well as the heterogeneity of the primary tumor. Undetectable ctDNA was associated with complete remission after surgery, but it was also found in tumors with no direct access to the CSF. Gene panels with 500 and 952 genes were used to analyze and compare tissue DNA with ctDNA obtained from CSF and plasma. Mutations detected in CSF were: KMT2D (32.0%), KMT2C (28.0%), SMARCA4 (24.0%), BCOR (20.0%), TP53 (12.0%), PTCH1 (8%), EP300 (8%), NF1 (8%), SETD2 (8%), MED12 (8%), SPEN (8%), CTNNB1 (4%), CREBBP (4%), PIK3CA (4%), LRP1B (4%), and FBXW7 (4%)^[76]^. Mutations detected in plasma were attributed to possible damage to the blood-brain barrier facilitating the entry of ctDNA into the bloodstream. CSF-ctDNA can predict disease progression and can detect more mutations than matched tissue. This may help in diagnosis, monitoring, and targeted therapy.

Lee and colleagues analyzed the CSF from medulloblastoma patients and identified metabolites, lipids, and cancer-specific RNAs for hypoxia, as well as cancer-specific RNAs^[70]^. Although subgrouping was challenging and not the primary goal, the study was able to reveal a group of omics signatures to separate cancer from normal CSF. A novel circular RNA, circ_463, as a sensitive biomarker for recurrence should be validated in further clinical studies.

Pagès and colleagues confirmed a major challenge of very low ctDNA levels in 258 pediatric CNS tumors of 13 different tumor types, mostly low-grade gliomas (*n* = 102), but also including almost 10% of embryonal tumors including medulloblastomas (*n* = 27)^[77]^. Harvesting ctDNA from CSF allowed the detection of CNAs in 20% and sequencing alterations in 30% of the samples, whereas plasma reached only detection sensitivities of 1.3% and 2,7%, respectively. Urine samples were all negative. Therefore, molecular profiling of ctDNA appears to be feasible for only a small subset of primary CNS tumors in children, such as medulloblastomas and other high-grade tumors. The low number of clonal aberrations in most medulloblastomas poses a challenge for the clinical application of sequencing methods.

Despite over 300 “medulloblastoma” studies listed on the clinicaltrials.gov webpage, only one includes “ctDNA” as a monitoring marker in blood and CSF. Therefore, most clinical studies on medulloblastomas focus on standard MRI and CSF cytology monitoring for measuring progression-free survival (PFS) and overall survival (OS).

## CONCLUSION

MRD-guided clinical decisions are now the standard of care for pediatric leukemia, sparing toxic therapy if not needed and identifying poor patient responses for more aggressive treatments. To achieve similar success in medulloblastoma management, sensitive and specific markers for the detection of MRD in medulloblastoma patients need to be confirmed. Recent progress in diagnostics and subtyping of medulloblastomas with risk stratification based on genetic profiling of primary tumor samples can reproducibly be applied to ctDNA as long as sufficient amounts can be isolated from body fluids of the patient. Gene alterations found in ctDNA such as TP53, PTCH1, MYCN, GLI2, SUFU, and 17p loss represent the original tumor and allow a less invasive molecular diagnosis and prognosis. Currently, CSF is the preferred source of ctDNA and appears to be much more sensitive than blood or urine. Varying but promising results can be obtained for early molecular diagnosis, even before the removal of the tumor. For some but not all patients, sequential analysis of ctDNA also allows close monitoring of tumor development after treatment. Compared to standard clinical monitoring by MRI and CSF cytology, ctDNA can detect MRD and relapse up to several months earlier, which opens a window for a better outcome. Early risk stratification should help the clinician to identify those patients who may benefit from aggressive chemo- and radiotherapy with the intent to cure as well as other patients who may not need it to protect them from unnecessary long-term damage including direct neurologic damage and secondary tumors. Ideally, CSF should be acquired shortly before and four weeks after the resection, followed by regular controls connected to clinical events. The clinical use of ctDNA analysis in medulloblastoma management can offer clear advantages over standard monitoring by MRI and cytology of CSF. The challenges of sensitivity to low amounts of ctDNA and artifacts are well defined and may be overcome with only small steps in technology improvement, including new devices and methods, and then providing the next gold standard. Fast and high-quality processing of CSF may improve the ctDNA quality and should be validated in larger clinical studies. In contrast, the potential use of CTC detection in medulloblastomas currently appears to be a much bigger challenge and most likely only restricted to highly specialized academic environments. Due to the low mutation rate in medulloblastomas, epigenetic markers, as well as specific circRNAs, should be included as markers for MRD in addition to classical mutation profiles. Ongoing progress in analytical methods, including proteomics and the potential role of EVs, as well as the reduction of artifacts, may offer new chances to establish liquid biopsy not only from smaller samples of CSF but also from blood or urine. After a century of milestones in neurosurgery, irradiation, and chemotherapy, the new molecular classification of medulloblastomas will progress with ctDNA from CSF as a promising biomarker for early diagnosis and better monitoring for improved clinical management of these childhood brain tumors.

## DECLARATIONS

### Copyright

© The Author(s) 2022.
